# Supplementary material for: Multicentre Surveillance of Candida Species from Blood Cultures during the SARS-CoV-2 Pandemic in Southern Europe (CANCoVEU Project)
Source: Microorganisms. 2023 Feb 23;11(3):560. doi: 10.3390/microorganisms11030560 (PMC10058953; doi:10.3390/microorganisms11030560)
Supplement: Supplementary file 1 [file microorganisms-11-00560-s001.zip › microorganisms-2229860-supplementary.pdf]

Table S1. Candida species identification and susceptibility results methods according to hospital center.

Hospital Centre	Country	Hospital beds	*Candida* species identification method	*Candida* species susceptibility results method	Clinical breakpoints interpretation
Città della Salute e della Scienza di Torino	Turin, Italy	1,900	MALDI Biotyper® Bruker	MICRONAUT-AM Antifungal Agents MIC, MERLIN Diagnostika GmbH Systems	EUCAST
Centro Hospitalar Universitário de Lisboa Central	Lisbon, Portugal	1,300	Vitek MS, Biomérieux	Etest, Biomérieux	EUCAST
Hospital Universitario La Paz	Madrid, Spain	1,200	MALDI Biotyper® Bruker	Sensititre™ YeastOne™, TREK Diagnostic Systems	CLSI
University Hospital of Ioannina	Ioannina, Greece	845	Vitek 2, Biomérieux	Vitek 2, Biomérieux	CLSI
Policlinico Universitario Paolo Giaccone	Palermo, Italy	530	MALDI Biotyper® Bruker	Sensititre™ YeastOne™, TREK Diagnostic Systems<br>Etest, Biomérieux<br>Disc diffusion, Liofilchem	CLSI
Nicosia General Hospital	Nicosia, Cyprus	450	Vitek 2, Biomérieux	Vitek 2, Biomérieux	EUCAST

Table S2. Proportion per year and comparison of distribution of *Candida* species isolates according to hospital ward.

Candida species	Emergency % (n)		Medical ward % (n)		Surgical ward % (n)	
	2020 n=13	2021 n=19	2020 n=142	2021 n=118	2020 n=76	2021 n=85
C. albicans	38.5 (5)	52.6 (10)	40.9 (58)	45.8 (54)	39.5 (30)	**56.4** (48)
C. parapsilosis complex	7.7 (1)	15.8 (3)	38.7 (55)	29.7 (35)	36.8 (28)	**20** (17)
C. glabrata	38.5 (5)	15.8 (3)	10.6 (15)	**20.3** (24)	13.2 (10)	15.3 (13)
C. tropicalis	15.3 (2)	10.5 (2)	7.7 (11)	2.5 (3)	7.9 (6)	5.9 (5)
C. krusei	-	5.3 (1)	-	1.7 (2)	-	-
C. guilliermondii	-	-	-	-	1.3 (1)	2.4 (2)
C. lusitaniae	-	-	0.7 (1)	-	1.3 (1)	-
C. dubliniensis	-	-	1.4 (2)	-	-	-

	ICU % (n)		COVID-19 ICU % (n)		COVID-19 ward % (n)	
	2020 n=103	2021 n=73	2020 n=38	2021 n=58	2020 n=8	2021 n=12
C. albicans	35.9 (37)	**50.7** (37)	57.9 (22)	41.4 (24)	50 (4)	58.3 (7)
C. parapsilosis complex	45.6 (47)	**23.3** (17)	26.3 (10)	34.5 (20)	25 (2)	16.7 (2)
C. glabrata	12.6 (13)	15.1 (11)	10.6 (4)	5.2 (3)	25 (2)	16.7 (2)
C. tropicalis	1 (1)	2.7 (2)	2.6 (1)	10.3 (6)	-	8.3 (1)
C. krusei	3 (3)	6.9 (5)	-	5.2 (3)	-	-
C. guilliermondii	1.9 (2)	-	-	3.4 (2)	-	-

C. lusitaniae	-	1.3 (1)	2.6 (1)	-	-	-
C. dubliniensis	-	-	-	-	-	-

Abbreviations: ICU: Intensive Care Unit. Numbers in bold indicate statistically significant difference (p≤0.05).

Table S3. Azole and echinocandin resistance per year according to hospital ward.

Antifungal resistance	Emergency % (n)		Medical ward % (n)		Surgical ward % (n)	
	2020	2021	2020	2021	2020	2021
Fluconazole % (n)	- (0/13)	15.8 (3/19)	8 (11/137)	10.3 (12/117)	6.8 (5/73)	14.3 (11/77)
Voriconazole % (n)	- (0/9)	6.3 (1/16)	3.8 (5/131)	1.9 (2/105)	4.2 (3/71)	7.7 (6/78)
Echinocandin % (n)	9.1 (1/11)	6.3 (1/16)	0.8 (1/121)	1.1 (1/95)	4.8 (3/62)	1.5 (1/66)
	ICU % (n)		COVID-19 ICU % (n)		COVID-19 ward % (n)	
	2020	2021	2020	2021	2020	2021
Fluconazole % (n)	24.7 (24/97)	23.2 (16/69)	5.3 (2/38)	14 (8/57)	- (0/8)	- (0/12)
Voriconazole % (n)	6.5 (6/92)	7.5 (5/67)	- (0/37)	1.9 (1/53)	- (0/8)	- (0/12)
Echinocandin % (n)	1.3 (1/79)	1.7 (1/58)	- (0/33)	5.8 (3/52)	- (0/7)	- (0/13)

Numbers in bold indicate statistically significant difference between 2020 and 2021 ($p \leq 0.05$).
